# Surgical Tips in Frozen Abdomen Management: Application of Coliseum Technique

**DOI:** 10.1155/2015/309290

**Published:** 2015-05-10

**Authors:** Ioannis D. Kyriazanos, Dimitrios K. Manatakis, Nikolaos Stamos, Christos Stoidis

**Affiliations:** 1st Surgical Department, Athens Naval and Veterans Hospital, 11521 Athens, Greece

## Abstract

Wound dehiscence is a serious postoperative complication, with an incidence of 0.5–3% after primary closure of a laparotomy incision, and represents an acute mechanical failure of wound healing. Relatively recently the concept of “intentional open abdomen” was described and both clinical entities share common pathophysiological and clinical pathways (“postoperative open abdominal wall”). Although early reconstruction is the target, a significant proportion of patients will develop adhesions between abdominal viscera and the anterolateral abdominal wall, a condition widely recognized as “frozen abdomen,” where delayed wound closure appears as the only realistic alternative. We report our experience with a patient who presented with frozen abdomen after wound dehiscence due to surgical site infection and application of the “Coliseum technique” for its definitive surgical management. This novel technique represents an innovative alternative to abdominal exploration, for cases of “malignant” frozen abdomen due to peritoneal carcinomatosis. Lifting the edges of the surgical wound upwards and suspending them under traction by threads from a retractor positioned above the abdomen facilitates approach to the peritoneal cavity, optimizes exposure of intra-abdominal organs, and prevents operative injury to the innervation and blood supply of abdominal wall musculature, a crucial step for subsequent hernia repair.

## 1. Introduction

Acute abdominal wound failure (also known as burst abdomen, evisceration, wound dehiscence, wound disruption, and fascial dehiscence) is a serious postoperative complication after primary closure of a laparotomy incision. It represents the unintentional creation of an open abdomen, with an incidence ranging between 0.5% and 3% of all laparotomies [1]. Wound breakdown may be complete, affecting all layers of the abdominal wall including the skin, or incomplete when the skin remains intact.

Recently the concept of intentional creation of a postoperative open abdomen, as a result of a deliberated therapeutic procedure in patients with specific intra-abdominal conditions, significantly differentiated the definition and management of “postoperative open abdominal wall” (POAW).

López-Cano et al. in a comprehensive review in 2013 presented the concept of “acute postoperative open abdominal wall” (acute POAW), considered as a unique nosological clinical entity resulting from intentional or unintentional surgery-related actions and composed by different interrelated clinicotherapeutical scenarios [1].

Although early reconstruction is the target, a significant proportion of POAW patients will develop adhesions between abdominal viscera and the undersurface of the anterior abdominal wall, a condition widely known as “frozen abdomen,” where delayed wound closure appears as the only realistic alternative [2].

Several operative techniques have been proposed for “frozen abdomen” management, indicating its exceptional characteristics and demanding nature. “Coliseum” technique, initially described by Sugarbaker, is an innovative alternative in abdominal cavity exploration, mainly for cases of peritoneal carcinomatosis [3]. We describe this technique in a patient with a “nonmalignant” frozen abdomen, developed after a postoperative abdominal wound disruption due to surgical site infection.

## 2. Case Presentation

A 45-year-old Caucasian male patient (BMI 38.2) underwent open appendectomy, through an oblique McBurney incision, for acute appendicitis in a district hospital. On the 2nd postoperative day, he became feverish and imaging reevaluation with abdominal CT scans demonstrated an abscess in the right iliac fossa. Exploratory relaparotomy through the same incision revealed appendiceal stump rupture, which was primarily sutured, after drainage of the abscess.

Despite his temporarily improved clinical condition, the patient was referred to our department on the 10th postoperative day when he developed mild pyrexia, with leukocytosis and elevated CRP. Physical examination revealed signs of surgical site infection. Abdominal and chest CT scans excluded pulmonary infection, intra-abdominal collection, or contrast medium extravasation around the cecum.

The surgical wound was explored under general anesthesia. Severe cellulitis and subcutaneous fat necrosis were found, along with a bulky mass of firmly adhered small bowel loops protruding through a wide postoperative hernia orifice. The wound was left open and a vacuum-assisted closure (VAC) device was applied for continuous wound drainage, while broad-spectrum antibiotics were empirically initiated.

Gradually the patient's general condition and trauma local parameters improved; however, 6-week application of VAC resulted in a large fascial defect. Protruding bowel loops caused intermittent episodes of intestinal obstruction, necessitating definitive, operative management.

During the third and final laparotomy, 12 weeks since the first operation, we decided to approach our case of “frozen abdomen” by the “Coliseum” technique. This includes lifting the edges of the surgical wound upwards and suspending them under traction by threads, thus optimizing exposure of intra-abdominal viscera.

**Figure 1:**
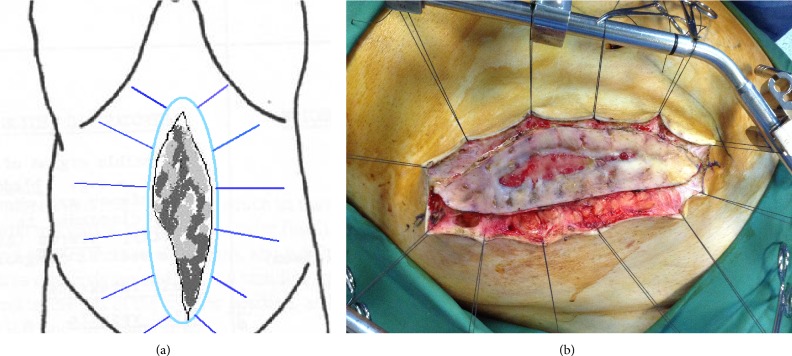
Elliptical incision, away from the granulating surface of the open abdominal wall.

**Figure 2:**
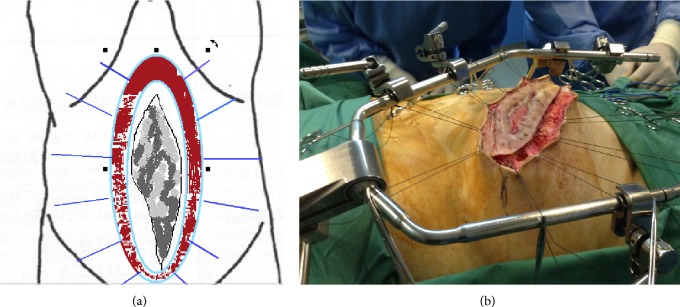
Edges of the surgical wound were lifted upwards and suspended under traction by threads from a frame positioned horizontally above the abdomen.

The operation commenced with a lateral elliptical incision to the skin, 2–4 cm away from the edges of the granulating tissue developed in POAW (Figure 1). The skin edges of the surgical wound were suspended under traction by threads from a frame positioned horizontally above the abdomen (Figure 2).

**Figure 3:**
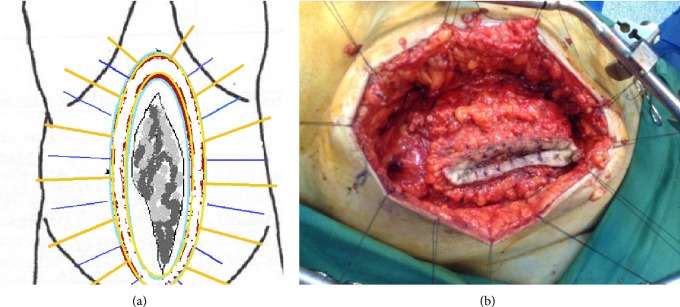
New elliptical incision and suspension of the aponeurotic leaf of external oblique abdominal muscle under traction. Lateralization of dissection and component separation technique application.

By deepening our dissection of the anterior abdominal wall through subcutaneous fatty tissue, we reached the aponeurosis of the external oblique muscle, in an area away from inflammatory changes and with healthy appearance. A second elliptical incision was performed in the aponeurosis, in a fashion similar to the skin, and a second row of stitches placed at the edges of this fascia incision permitted suspension of the aponeurotic leaf under traction (Figure 3). Lateralization of aponeurosis dissection bilaterally ensured identification of the lateral edges of both rectus abdominis muscles and, after performing relaxing incisions, permitted application of component separation technique for the coverage of the abdominal wall defect (Figure 3).

**Figure 4:**
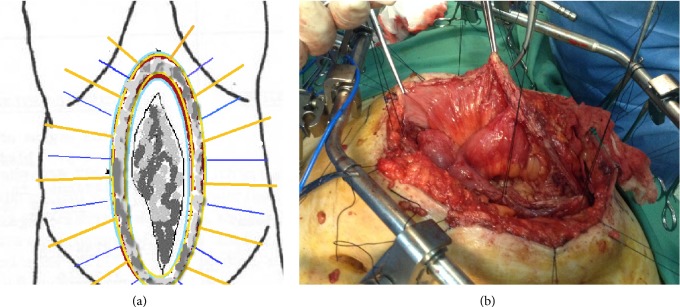
Third row of suspension threads was placed on the peritoneum, providing wide exposure of the peritoneal cavity. Isolation of the abdominal viscera involved in “frozen abdomen.”

Next, the peritoneum was incised and the abdominal cavity entered. A third row of suspension threads was placed on the peritoneum, providing wide exposure of the peritoneal cavity and isolating the abdominal viscera involved in “frozen abdomen” (Figure 4). The cecum and a significant length of terminal ileum were involved in frozen abdomen formation. As expected, dissection of the involved viscera without causing bowel perforation was judged virtually impossible, a result that would preclude the use of prosthetic mesh for hernia repair. The right hemicolon, the last 60 cm of ileum, and the involved part of the anterior abdominal wall including the old scar were resected finally en bloc.

In combination with component separation technique, which permitted the approximation of anterior abdominal wall musculature and aponeurotic layers, a double-layer mesh was placed intra-abdominally and fixed with nonabsorbable sutures for definitive hernia defect repair.

The patient's postoperative course was uneventful and he was discharged on the 7th postoperative day. He remains free of symptoms at the 12-month follow-up.

## 3. Discussion

Surgical wound dehiscence has been described for many years and remains a serious postoperative complication, carrying significant morbidity and mortality. Recently characterized with the term “unintentional acute postoperative open abdominal wall,” it denotes an acute mechanical failure of wound healing [1].

Among several risk factors [4] wound contamination is the single most important for abdominal wound disruption [5], with a risk score of 1.9, as van Ramshorst et al. have shown [6, 7]. Our patient, being obese and relatively malnourished after two sequential laparotomies, was at increased risk for developing dehiscence, due to local peritonitis and surgical site infection.

Despite the reasons prompting its development and once the therapeutic objective has been achieved, as early as possible closure of the abdominal wall represents the target. High morbidity rates (34%–44%), with prolonged hospital stay and increased cost, underline the necessity for appropriate treatment [8]. The optimal strategy is still a matter of debate, as there are no data from randomized controlled studies. Important determinators of therapeutic decision include (a) presence of wound contamination, (b) fixation of abdominal viscera to the anterolateral abdominal wall, and (c) presence of enteroatmospheric fistula. POAW can be accordingly categorized as follows: (1) POAW without fixation (1A: clean, 1B: contaminated, and 1C: with bowel leak), (2) POAW with developing fixation (2A: clean, 2B: contaminated, and 2C: with bowel leak), (3) POAW with developed fixation (frozen abdomen) (3A: clean frozen abdomen, 3B: contaminated frozen abdomen), and (4) POAW with established frozen abdomen and enteroatmospheric fistula [2, 9].

Our patient presented to us with developing fixation, contaminated dehisced wound (class 2B). These cases are best treated as open abdomen, to prevent abdominal compartment syndrome and to control local inflammation in the short term, resulting in decreased incidence of frozen abdomen and need for planned hernia management in the long term [10–12].

Improvement of local wound conditions allows for an attempt at definitive closure of the musculofascial layers, but in a very limited time frame of 2-3 weeks preferably during the same hospitalization [2]. Prolongation of expectant management (>3 weeks) due to inadequate improvement leads to creation of “frozen abdomen” with a planned incisional hernia repair being the only realistic treatment alternative [1].

Our choice of VAC closure offered improvement of local wound conditions and patient's general status but prolonged the total waiting period to 12 weeks from POAW identification resulting in class 3A POAW with increased bowel fixation and loss of domain due to further fascial lateralization making tension-free repair more difficult [13–15].

Recurrent episodes of small bowel obstruction prompted our operative intervention earlier than scheduled as “obliterative peritonitis” in “frozen abdomen” cases takes at least 4 months to subside and allow safe laparotomy and adhesiolysis [15].

The main goal during “frozen abdomen” surgical management is to approach the peritoneal cavity through laterally placed incisions, away from the granulating tissue to prevent bowel trauma and contamination of the surgical field, which jeopardizes the use of mesh [16]. Moreover, in the more elaborate cases of enteroatmospheric fistulae, where bowel resection is practically unavoidable, the extent of resection should be kept to a minimum to avoid the possibility of short bowel syndrome [17].

Demetriades reported an approach to the abdominal cavity via long vertical incisions located 8–10 cm laterally to the open abdominal wound and mobilization of the adhered bowel loops under direct vision from lateral towards the midline. The defect was covered by a crystalline polypropylene and high-density polyethylene Marlex mesh and the skin and subcutaneous tissue were closed over the mesh [18].

Sriussadaporn et al. described entering the abdomen with an incision around the granulating tissue of the scar. After excision of the involved enteric loop and enteroenteric anastomosis, the abdominal defect was closed with an absorbable sterile mesh constructed of undyed filaments prepared from a homopolymer of glycolic acid. The material, which is inert, noncollagenous, and nonantigenic, was subsequently covered with bilateral bipedicled anterior abdominal skin flaps [19].

Marinis et al. reported their experience with several cases of “frozen abdomen” with enterocutaneous fistulae and proposed a relatively early intervention, based on a lateral surgical approach via the circumference of the POAW. To take down the fistula, an enterectomy of the associated enteric loop was performed and the abdominal defect was closed by an absorbable mesh. The surgical wound was either left open to granulate or a VAC device was applied [20].

“Coliseum” technique represents an innovative alternative to abdominal exploration, for cases of “malignant” frozen abdomen due to peritoneal carcinomatosis [3]. By lifting the edges of the surgical wound upwards and suspending them under traction by threads from a frame positioned horizontally above the abdomen, a large space in continuity with the abdominal cavity is created (peritoneal expansion), optimizing exposure of intra-abdominal organs for hyperthermic intraperitoneal chemotherapy administration [21].

In our patient, this maneuver facilitated the approach to the peritoneal cavity not directly through the POAW, but rather with laterally placed incisions away from the granulating surface. Furthermore, constant traction on the edges of the abdominal incision elevated the layers of the abdominal wall and facilitated their accurate dissection, also preventing operative injury to the innervation and blood supply of the muscles, a crucial step in the possibility of subsequent application of “component separation technique” [22].

Suspension threads provide wide exposure of the peritoneal cavity, while simultaneously isolating the part of abdominal wall and content (bowel loops) involved in “frozen abdomen.” Additionally, this kind of wide abdominal wall components dissection facilitated mesh placement after bowel resection and anastomosis.

## 4. Conclusion

We describe the use of the Coliseum technique in order to approach a “nonmalignant” frozen abdomen developed after a postoperative abdominal wound disruption due to infection and contamination. The use of this “serially applied suspending Coliseum technique” creates constant traction to all dissected abdominal wall layers, isolates the adhered “frozen abdomen,” and creates a wide operating field in convenience of the surgeon. We believe that this “oncosurgical” operative approach can be safely and efficiently applied also in “benign” frozen abdomens increasing the interventional technical alternatives.
